# Shexiang Tongxin Dripping Pills regulates SOD/TNF-α/IL-6 pathway to inhibit inflammation and oxidative stress to improve myocardial ischemia-reperfusion injury in mice

**DOI:** 10.3389/fcvm.2025.1571925

**Published:** 2025-06-12

**Authors:** Wanying Du, Chenguang Zhai, Huijie Zhang, Jun Ren, Xiaoyang Chen, Xuejing Sun, Chun Li, Wei Wang, Yijun Chen

**Affiliations:** ^1^ College of Basic Medicine, Guangzhou University of Chinese Medicine, Guangzhou, Guangdong, China; ^2^ Guangdong Key Laboratory of Institute of Formula and Syndrome, Guangzhou, Guangdong, China; ^3^ College of Traditional Chinese Medicine, Guangzhou University of Chinese Medicine, Guangzhou, Guangdong, China; ^4^ Traditional Chinese Medicine Guangdong Laboratory (TCM-GL), Zhuhai, Guangdong, China

**Keywords:** Shexiang Tongxin Dropping Pills, ischemia reperfusion injury, oxidative stress, inflammatory factors, cardiac function

## Abstract

**Introduction:**

Shexiang Tongxin Dropping Pills (STDP), a traditional Chinese medicine (TCM), is clinically used for cardiovascular diseases like myocardial ischemia. Myocardial ischemia-reperfusion injury (MIRI), worsened by oxidative stress and inflammation, remains a significant problem, and the mechanisms underlying STDP's cardioprotection are incompletely understood. This study aimed to investigate STDP's effects on the SOD/TNF-α/IL-6 pathway and its impact on inflammation and oxidative stress in MIRI.

**Methods:**

A mouse model of MIRI was employed to evaluate the cardioprotective effects and mechanisms of STDP *in vivo*. Pretreatment with STDP was administered prior to MIRI induction. Assessments included serum SOD activity, cardiac tissue ROS levels, cardiomyocyte apoptosis rates (TUNEL assay), mRNA and protein expression of IL-1β, TNF-α, and IL-6 (qPCR, Western blot), histopathological evaluation of myocardial tissue morphology and inflammatory infiltration (H&E staining), myocardial infarction size (TTC staining), and cardiac function parameters (contractility, diastolic function).

**Results:**

STDP pretreatment significantly enhanced serum SOD activity and reduced cardiac ROS levels and cardiomyocyte apoptosis. It effectively downregulated mRNA and protein expression of IL-1β, TNF-α, and IL-6. Histopathology revealed reduced inflammatory cell infiltration and more intact cardiomyocyte morphology in STDP-treated groups. TTC staining confirmed a reduction in myocardial infarction size. Cardiac function assessments showed STDP improved both contractility and diastolic function post-MIRI and reduced arrhythmia incidence.

**Discussion:**

STDP ameliorates MIRI in mice by inhibiting inflammatory responses and oxidative stress, primarily through modulation of the SOD/TNF-α/IL-6 pathway. Its cardioprotective effects include reducing apoptosis, inflammation, ROS, infarction size, and arrhythmias, while improving cardiac function and tissue repair. These findings elucidate a key mechanism for STDP and provide empirical support for its clinical use in MIRI, offering innovative perspectives for managing cardiovascular disorders with TCM and facilitating the integration of traditional and modern medicine.

## 1 Introduction

Acute myocardial infarction (AMI) represents a significant global health concern, marked by elevated incidence and mortality rates (1). Current therapeutic approaches for myocardial revascularization encompass percutaneous coronary intervention, coronary artery bypass grafting, and conventional thrombolytic therapy, all of which are capable of partially restoring blood perfusion to the affected myocardial region (2). Nevertheless, these interventions may inadvertently enhance oxygenated blood flow to the area surrounding the infarction, potentially resulting in myocardial reperfusion injury (MIRI) (3). Presently, there exists a considerable deficiency in safe and effective pharmacological agents aimed at alleviating MIRI (4).

Shexiang Tongxin Dropping Pills (STDP), which are derived from “Zhibaodan” in the “Taiping Huimin Heji Ju Fang” of the Song Dynasty, comprise seven components: artificial musk, total saponins from ginseng stems and leaves, toad venom, salvia miltiorrhiza, artificial bezoar, bear bile powder, and borneol. These constituents are recognized for their aromatic properties that benefit cardiac function, promote qi and blood circulation, alleviate meridian obstructions, and relieve pain. Currently, STDP are clinically employed for the management of stable angina pectoris associated with coronary heart disease (5). Contemporary pharmacological investigations have revealed that STDP possess anti-inflammatory, antiplatelet aggregatory, and antioxidant stress properties (6), which align with the fundamental pathophysiology of coronary heart disease and MIRI (7, 8). Recent studies have demonstrated musk's neuroprotective and anti-inflammatory properties (9). It has been shown to inhibit neuronal apoptosis and reduce oxidative stress, suggesting its potential role in treating neurological disorders such as stroke and Alzheimer's disease (10). Additionally, musk has been reported to enhance the penetration of other drugs across the blood-brain barrier, making it a valuable adjuvant in the formulation of neuroactive compounds (11). Borneol has been extensively studied for its ability to enhance the bioavailability and penetration of drugs across biological membranes (12). It has been shown to improve the absorption and distribution of various compounds, including those targeting the central nervous system (13). The active compounds in Ginseng, such as ginsenosides, have been shown to regulate blood pressure, improve heart function, and reduce oxidative stress (14). Ginseng has also been reported to enhance cognitive function and improve mood, making it a potential treatment for stress-related disorders (15). Salvia miltiorrhiza is a traditional Chinese medicine with a long history of use in cardiovascular diseases. Recent research has focused on its ability to inhibit platelet aggregation, reduce inflammation, and promote angiogenesis (16, 17). These properties make salvia miltiorrhiza a promising candidate for the treatment of cardiovascular diseases, including myocardial infarction, atherosclerosis, and stroke (18, 19). Bear bile powder has been traditionally used in Traditional Chinese Medicine for its detoxifying and bile-promoting properties. Modern research has delved into its anti-inflammatory, antioxidant, and hepatoprotective activities (20, 21). Bezoar, derived from the gallstones of certain bovines, has been extensively studied for its anti-inflammatory, antipyretic, and anticonvulsant properties (22). Its active components, including bilirubin and bile acids, have shown promise in treating febrile conditions, seizures, and inflammation-related disorders (23, 24). The bufadienolides present in toad venom have demonstrated efficacy in enhancing heart contractility and reducing edema (25). Furthermore, research has explored its potential as an anti-cancer agent, targeting specific signaling pathways involved in tumorigenesis (26). These findings underscore toad venom's therapeutic potential in cardiovascular diseases and oncology (27). However, the protective effects and underlying mechanisms of STDP on myocardial cells and blood vessels following MIRI remain inadequately understood.

The advent of interdisciplinary fields such as computational biology, bioinformatics, artificial intelligence, and big data science has facilitated the emergence of network pharmacology, offering a novel framework for elucidating the mechanisms of traditional Chinese medicine (TCM). By employing a “network” approach to regain a “holistic” perspective, significant progress has been achieved in medical research (28). Numerous studies have indicated that STDP can effectively diminish the accumulation of reactive oxygen species (ROS) in tissues, enhance superoxide dismutase (SOD) activity (29–31), and markedly reduce serum levels of tumor necrosis factor-α (TNF-α), interleukin-6 (IL-6), and interleukin-1β (IL-1β) in patients with heart failure. During episodes of myocardial ischemia-reperfusion, there is a substantial buildup of ROS, accompanied by a significant release of lactate dehydrogenase (LDH) into the bloodstream and a reduction in SOD levels, which exacerbates myocardial cell injury post-reperfusion (32–34). Concurrently, the accumulation of inflammatory cytokines such as IL-6, IL-1β, and TNF-α intensifies the inflammatory response, further worsening myocardial damage and exacerbating oxidative stress injury (35, 36).

Consequently, this study aims to explore the protective effects of STDP against myocardial ischemia-reperfusion injury by administering it prior to the induction of MIRI in a murine model. Building upon this foundation, we conducted a network pharmacological analysis of the *in vivo* components (37) to identify potential regulatory targets and pathways. Ultimately, we validated the core targets through methodologies such as enzyme-linked immunosorbent assay (ELISA) and Western blotting (WB) to elucidate the regulatory mechanisms by which STDP intervenes in MIRI.

## 2 Materials and methods

### 2.1 Animals

A total of sixty healthy male ICR mice, aged 8 weeks and weighing between 22 and 24 grams, classified as SPF grade, were procured from Guangdong Vital River Laboratory Animal Technology Co., Ltd. [License No.: SYXK (Guangdong) 2019–0202]. Following their acquisition, the mice were housed in individually ventilated cages within a SPF barrier system at the Experimental Animal Center of Guangzhou University of Chinese Medicine, which operates under a usage license number of SYXK (Guangdong) 2018-0085, in compliance with established animal experimentation standards. The breeding environment was maintained at a temperature range of 22–25°C, with relative humidity levels between 50% and 70%, and a 12-hour light/dark cycle. The mice were allowed a one-week acclimatization period prior to the commencement of the experiment and were housed individually with unrestricted access to food and water. The animal care and experimental protocols received approval from the Animal Ethics Committee of Guangzhou University of Chinese Medicine (Ethics No.: 20240721007), and the mice were treated with humane consideration in accordance with the principles of the 3Rs throughout the duration of the study.

### 2.2 Drugs

The Shexiang Tongxin Dropping Pills (STDP) were procured from the Shenglong Branch of Inner Mongolia Kangenbei Pharmaceutical Co., Ltd. (Batch No.: 190310). The Aspirin Enteric-coated Tablets (Aspirin) were acquired from Bayer Healthcare Co., Ltd. (Batch No.: 85551191).

### 2.3 Reagents

The interleukin (IL)-1β protein antibody and horseradish peroxidase (HRP) secondary antibody were acquired from Cell Signaling Technology, Inc. (USA), with catalog numbers 12703S and 7074S, respectively. Superoxide dismutase (SOD) and reactive oxygen species (ROS) assay kits were obtained from Beyotime Biotechnology (Shanghai) Co., Ltd., catalog numbers S0101S and S0033S, respectively. Enzyme-linked immunosorbent assay (ELISA) kits for interleukin-6 (IL-6) and tumor necrosis factor-alpha (TNF-α) were sourced from Suzhou Sipure Biotechnology Co., Ltd., with batch numbers 4AC052413 and 4AD222414, respectively. The SF594 TUNEL Cell Apoptosis Detection Kit (red fluorescence), Lactate Dehydrogenase (LDH) Activity Assay Kit, and Hematoxylin-Eosin (HE) Staining Kit were purchased from Beijing Solarbio Science & Technology Co., Ltd., with batch numbers T2195, BC0685, and G1120, respectively. The 2,3,5-triphenyltetrazolium chloride powder was obtained from Sigma-Aldrich (USA) with batch number T8877. Additionally, the RNA extraction kit, reverse transcription kit, and fluorescence quantitative kit were purchased from Yisheng Biotech Co., Ltd., with batch numbers 19211ES60, 11142ES10, and 11211ES03, respectively. Tribromoethanol was purchased from Nanjing Aibei Biotechnology Co., Ltd., catalog number M2910.

### 2.4 Instruments

The VS200 research-grade whole slide scanning system, specifically the laser slide panoramic scanner, was procured from Olympus (China). The EG1150C embedding machine and the SM2000R paraffin sliding microtome were acquired from Leica Microsystems GmbH (Germany). The VO-53 vacuum dryer was obtained from Shanghai Titan Scientific Co., Ltd. The Revos dehydrator was sourced from Thermo Fisher Scientific (USA). The NANODROP 2000 spectrophotometer and the QuantStudio™ 1 Plus real-time fluorescent quantitative PCR system were acquired from Thermo Fisher Scientific (Shanghai) Instruments Co., Ltd. The BL-420N signal acquisition and processing system was sourced from Chengdu Taimeng Software Co., Ltd. The Vinno 6lab small animal ultrasound instrument was obtained from Feino Technology Co., Ltd. The JB-P5 embedding machine and the JB-L5 freezing platform were sourced from Wuhan Junjie Electronics Co., Ltd. The Leica HistoCore AUTOCUT hard tissue microtome and the KD-P tissue spreading machine were procured from Zhejiang Kedi Instrument and Equipment Co., Ltd. The XSP-C204 microscope was obtained from Chongqing Liuhui Technology Co., Ltd. The small animal ventilator was sourced from Shanghai Aoke Biotechnology Co., Ltd. The Orbital Shaker TS-1 shaker was acquired from Haimen Qilin Bell Instrument Manufacturing Co., Ltd. The KZ-Ⅲ-FP low-temperature tissue homogenizer was obtained from Wuhan Saiweier Biotechnology Co., Ltd. The Synergy H1 microplate reader was sourced from BioTek Instruments, Inc. (USA). The JIDI-17R micro high-speed refrigerated centrifuge was procured from Guangzhou Jidi Instrument Co., Ltd. Finally, the Sartorius BP211D electronic analytical balance was obtained from Sartorius AG (Germany).

### 2.5 Methods

#### 2.5.1 Animal grouping

A total of sixty ICR mice were randomly assigned to one of five groups: a sham group, a model group, a positive control group, and three groups receiving low, medium, and high doses of STDP, with each group comprising ten mice.

#### 2.5.2 Model preparation

Thirty minutes following the final gavage, mice assigned to the model, positive drug, and STDP groups were subjected to myocardial ischemia-reperfusion injury modeling, whereas those in the sham group underwent thoracotomy without coronary artery ligation. In accordance with previously established protocols by our research team (38), the mice were anesthetized via intraperitoneal injection of 1% pentobarbital sodium (60 mg/kg), positioned supinely, shaved, placed on a temperature-controlled heating pad, and connected to an electrocardiogram (ECG) monitor. Tracheal intubation was performed orally to facilitate ventilation. Utilizing a dissecting microscope, the skin was prepared, and the pectoral muscles were separated bluntly in layers. The thoracic cavity was accessed between the fourth and fifth intercostal spaces, and the left anterior descending coronary artery was ligated 2 mm distal to the left auricular appendage using a 6–0 surgical suture. In the sham group, the suture was passed through but not tied. Myocardial ischemia was confirmed by the observation of a pale area on the cardiac surface and an ST-segment elevation or depression of 0.1 mV on the ECG. Following a 30-min ischemic period, reperfusion was initiated, with successful reperfusion indicated by a 50% reduction in the elevated ST-segment. The thoracic cavity was cleared of blood, and the chest wall was sutured in layers. After the resumption of spontaneous respiration, the ventilator was removed, and samples were collected for various analyses 24 h post-reperfusion.

#### 2.5.3 Drug administration

The clinical dose of STDP is 3.5 mg/kg/d. According to the transformation clinical formula equivalent doses for mice (mg/kg) = 0.13 × the dose for human (mg/kg) × 60 kg (5), we adjusted the dose administered to mice to 57.60 mg/kg/day as high doses, 27.30 mg/kg/day as middle doses and 13.65 mg/kg/day as low doses. STDP was dissolved in 1% sodium carboxymethyl. Seven days prior to the modeling phase, mice assigned to the low, medium, and high-dose STDP groups were administered 13.65 mg/kg, 27.30 mg/kg, and 57.60 mg/kg of STDP, respectively, via gavage. The positive control group was given 13.00 mg/kg of enteric-coated aspirin tablets. Meanwhile, both the sham and model groups received an equivalent volume of distilled water through gavage once daily for a duration of seven consecutive days.

#### 2.5.4 Echocardiographic assessment of mouse cardiac function

Mice were subjected to anesthesia and secured on the surgical table. Following the stabilization of respiratory and cardiac rates, M-mode echocardiography was employed to assess the left ventricular internal diameters at both end-systole and end-diastole from the parasternal short-axis perspective. The ultrasound system subsequently computed the left ventricular ejection fraction and left ventricular fractional shortening automatically.

#### 2.5.5 The mice serum and cardiac tissue samples collected

Mice were humanely euthanized via intraperitoneal injection of tribromoethanol (Avertin) at a dosage of 30 μl/g body weight. Prior to injection, the injection site was sterilized with 70% ethanol. Following administration, animals were monitored for the loss of righting reflex and response to nociceptive stimuli. Upon achieving full anesthesia, exsanguination was performed by carefully enucleating the eyeball to allow blood drainage into pre-labeled 1.5 ml Eppendorf tubes, followed by cervical dislocation to ensure complete euthanasia. Tubes were maintained at room temperature for 30–60 min to facilitate coagulation. After clot formation, samples were centrifuged at 3,000 rpm for 10 min to separate serum from cellular components. Serum was carefully aspirated and transferred into sterile cryovials for storage at −80°C until subsequent use. For tissue harvesting, midline thoracotomy was performed using sterile surgical instruments. Hearts were meticulously dissected and rinsed with ice-cold phosphate-buffered saline (PBS) to remove residual blood and contaminants. For cryopreservation, cardiac tissues were directly placed into pre-labeled cryovials, snap-frozen in liquid nitrogen, and promptly transferred to −80°C freezer. Alternatively, for histological preservation, hearts were immersed in freshly prepared 4% paraformaldehyde in PBS solution with sufficient volume. Tissues were fixed at room temperature for 24 h with gentle agitation.

#### 2.5.6 Cardiac tissue processing and TTC staining protocol for myocardial infarction quantification

Hearts from euthanized mice underwent systemic perfusion with cold saline to remove residual blood components. Excised hearts were briefly rinsed in PBS and trimmed before immediate embedding in OCT compound within pre-chilled (−80°C) cryomolds. The embedded tissues were flash-frozen in liquid nitrogen-chilled isopentane and stored at −20°C for subsequent analysis. For histological evaluation, 90% of collected specimens were sectioned coronally at 2-mm intervals using a precision cryostat. Tissue sections underwent vital staining with 2% 2,3,5-triphenyltetrazolium chloride (TTC, Sigma-Aldrich) in PBS (pH 7.4) for 20 min at 37°C, followed by overnight fixation in 4% neutral buffered formalin. The stained sections were digitally photographed under standardized illumination conditions. Myocardial infarction area was quantitatively analyzed using ImageJ software (NIH v1.53) by calculating the percentage of TTC-negative (infarcted) tissue relative to total left ventricular area across all sections.

#### 2.5.7 He staining and pathological morphological observation

Myocardial tissue obtained from the ischemic central region was preserved in 4% neutral buffered formalin, subsequently embedded in paraffin, and sectioned to a thickness of 4 μm. The sections were stained with hematoxylin and eosin (HE) and analyzed using the Olympus VS200 whole slide scanning system to identify appropriate fields of view.

#### 2.5.8 Immunofluorescence detection of ROS expression

Frozen myocardial tissue sections were subjected to incubation with the DCFH-DA probe at 37°C for a duration of 30 min in a dark environment. This was followed by a 10-min DAPI staining of the cell nuclei, also conducted in the dark. Subsequent to the mounting process, the slides were photographed and analyzed for fluorescence signal intensity utilizing the VS200 research-grade whole slide scanning system, specifically employing a laser slide panoramic scanner with an excitation wavelength of 488 nm and an emission wavelength of 525 nm. The reactive oxygen species (ROS) staining exhibited a green fluorescence, while the DAPI staining displayed a blue fluorescence. The relative fluorescence intensity was quantified using ImageJ software, with the calculation for ROS levels expressed as a percentage: ROS level (%) = (green fluorescence intensity/blue fluorescence intensity) × 100%.

#### 2.5.9 Detection of myocardial cell apoptosis in mice groups via TUNEL staining

Frozen myocardial tissue sections were thawed at room temperature for 15 min to eliminate ice crystals. The TUNEL staining procedure was performed according to the manufacturer's instructions (Solarbio). The sections were fixed with 4% paraformaldehyde (prepared in PBS) at room temperature for 30 min, followed by two 10-minute washes with PBS. Excess liquid around the sections was absorbed with filter paper, and the sample outlines were marked with an immunohistochemical pen to facilitate subsequent permeabilization and labeling. Proteinase K solution (2 mg/ml) was diluted with PBS at a ratio of 1:100 to a final concentration of 20 µg/ml. Each sample was covered with 100 µl of the diluted solution and incubated at 37 ℃ for 120 min. The sections were then washed twice with PBS for 5 min each, and excess liquid was removed with filter paper. The processed samples were placed in a humidified chamber to maintain moisture. TUNEL reaction mixture was freshly prepared and added to each sample (50 µl per sample) to ensure even coverage. The samples were incubated at 37 ℃ in the dark for 2 h. The TUNEL reaction mixture was discarded, and the sections were washed twice with PBS, followed by three 5-minute washes with 0.1% Triton X-100 (prepared in PBS containing 5 mg/ml BSA) to remove unbound labeling molecules. The sections were dried, and anti-fade mounting medium containing DAPI was applied for coverslipping. The mounted sections were scanned using the VS200 research-grade whole-slide scanning system. The excitation and emission wavelengths were set at 594 nm and 615 nm for red fluorescence, and 358 nm and 461 nm for DAPI, respectively. Appropriate exposure times and gain settings were applied to obtain clear fluorescent images. The acquired fluorescent images were opened using the VS200-integrated image analysis software. Suitable analysis regions were selected to ensure consistency and representativeness. The intensities of red (TUNEL) and blue (DAPI) fluorescence were measured using the measurement tool in ImageJ software, with correct fluorescence channels and measurement areas selected. The relative fluorescence intensity was calculated using the formula: TUNEL level (%) = red fluorescence intensity/blue fluorescence intensity×100%, which was used to evaluate the proportion of TUNEL levels in myocardial tissue relative to the number or size of nuclei.

#### 2.5.10 RT-qPCR detection of TNF-α, Il-6, and Il-1β mRNA expression

Primers targeting TNF-α, IL-6, and IL-1β mRNA were developed based on gene sequences obtained from the Gene Bank and subsequently synthesized by Sangon Biotech (Shanghai) Co., Ltd. (refer to Table 1 for primer sequences). Cardiac tissue was meticulously dissected into small fragments on ice, homogenized, and total RNA was extracted utilizing the Trizol method. Reverse transcription to complementary DNA (cDNA) was conducted in accordance with the manufacturer's instructions (conditions: 37℃ for 15 min, 85℃ for 5 s, followed by a hold at 4℃). Quantitative fluorescent analysis was executed following the guidelines of the TaqMan multiplex qPCR master mix kit (conditions: initial denaturation at 95℃ for 5 min, succeeded by 45 cycles of denaturation at 95℃ for 5 s and annealing/extension at 60℃ for 30 s). The relative expression levels in each group were determined using the 2^−*ΔΔ*Ct^ method.

**Table 1:** The list of specific primers used for qRT-PCR.

Gene	Primer (5′ to 3′)	
IL-1β	GAAATGCCACCTTTTGACAGTG（F）	TGGATGCTCTCATCAGGACAG(R)
TNF-α	CAGGCGGTGCCTATGTCTC（F）	CGATCACCCCGAAGTTCAGTAG(R)
IL-6	CTGCAAGAGACTTCCATCCAG（F）	AGTGGTATAGACAGGTCTGTTGG(R)
GAPDH	AGGTCGGTGTGAACGGATTTG（F）	GGGGTCGTTGATGGCAACA(R)

F, forward primer; R, reverse primer.

#### 2.5.11 Acquisition of drug targets

The relevant targets for the candidate compounds and diseases were determined by conducting searches in several databases, including PubChem (https://pubchem.ncbi.nlm.nih.gov/) (39), BATMAN-TCM (http://bionet.ncpsb.org.cn/batman-tcm/) (40), and GeneCards (https://www.genecards.org/) (41). Instances of duplicate targets were eliminated, as it is common for multiple compounds or diseases to be associated with several targets.

#### 2.5.12 Network construction and analysis

The intersection of targets associated with specific compounds and diseases was determined utilizing the Venny version 2.1.0. The protein-protein interaction (PPI) network was developed using the STRING database (https://cn.string-db.org/) (42). Additionally, the compound-target-disease network was established through the use of Cytoscape version 3.10.2. A comprehensive analysis of the network's topological properties was performed employing the CytoNCA plugin within Cytoscape. The PPI network was constructed on the STRING database platform, followed by a topological analysis aimed at elucidating the target interaction network and identifying key targets.

#### 2.5.13 Validation of core targets in key pathways enriched in section 2.9 and measurement of indicators

Blood samples were obtained from the ocular region 24 h following reperfusion and subjected to centrifugation at 3,000 revolutions per minute for a duration of 10 min. The serum was subsequently collected and analyzed for levels of lactate dehydrogenase (LDH), superoxide dismutase (SOD), tumor necrosis factor-alpha (TNF-α), and interleukin-6 (IL-6) in accordance with the protocols provided by the respective LDH and SOD assay kits, as well as the ELISA kits for TNF-α and IL-6. Approximately 20 mg of myocardial tissue from mice was excised, minced, and placed in a homogenizer containing 0.2 ml of RIPA protein lysis buffer, which included protease inhibitors, phosphatase inhibitors, and phenylmethylsulfonyl fluoride (PMSF), to facilitate thorough homogenization. The homogenate was allowed to incubate on ice for 30 min and was then sonicated in an ice-water bath for 10 min to ensure complete lysis of the tissue. The lysate was transferred to a 1.5 ml centrifuge tube and centrifuged at 4℃ at 12,000 revolutions per minute (with a centrifugal radius of 40 cm) for 15 min. The supernatant was collected, and the protein concentration of each sample was quantified using the bicinchoninic acid (BCA) method, followed by adjustment to achieve uniformity across samples. An equal volume of protein loading buffer was incorporated, and the mixture was heated at 95℃ for 5 min to denature the proteins. A volume of 10 μl of the protein sample was then applied to SDS-PAGE gel electrophoresis, followed by membrane transfer, antigen blocking, primary antibody incubation, and secondary antibody incubation, among other procedural steps. Finally, enhanced chemiluminescence (ECL) color development solution was utilized for visualization, and images were captured using a gel imager. The gray value of the β-actin protein band served as an internal reference, and the relative expression levels of the target protein were analyzed using Image J software.

#### 2.5.14 Statistical methods

All statistical analyses were performed using GraphPad Prism software (version 8.0.2) (43). Datasets were imported into the software and evaluated for distribution properties via the *Normality and Lognormality Tests* module under column analyses. This assessment determined compliance with Gaussian (normal) or lognormal distributions and calculated relative sampling probabilities from these distributions (assuming no alternative distributions). Normality testing incorporated three complementary methods: the Shapiro–Wilk test (44), Kolmogorov–Smirnov test (45) with Lilliefors correction, and the D'Agostino-Pearson omnibus test. A significance threshold of *α* = 0.05 was applied, with *P* ≥ 0.05 indicating adherence to a normal distribution. For normally distributed data, intergroup comparisons were conducted using one-way analysis of variance (ANOVA) (46). When ANOVA revealed significant between-group variance (*P* < 0.05), *post hoc* pairwise comparisons of means were performed using Tukey's Honest Significant Difference (HSD) test (47) to control family-wise error rates. Non-normally distributed datasets were analyzed via the Kruskal–Wallis nonparametric test, followed by Dunn's multiple comparisons test (48) with Bonferroni adjustment. Specific analytical objectives included: ① Comparisons between experimental groups and the model control group. ② Dose-dependent effects across STDP treatment groups.

## 3 Results

### 3.1 Effects of Shexiang Tongxin Dropping Pills on cardiac function in mice

**Figure 1:**
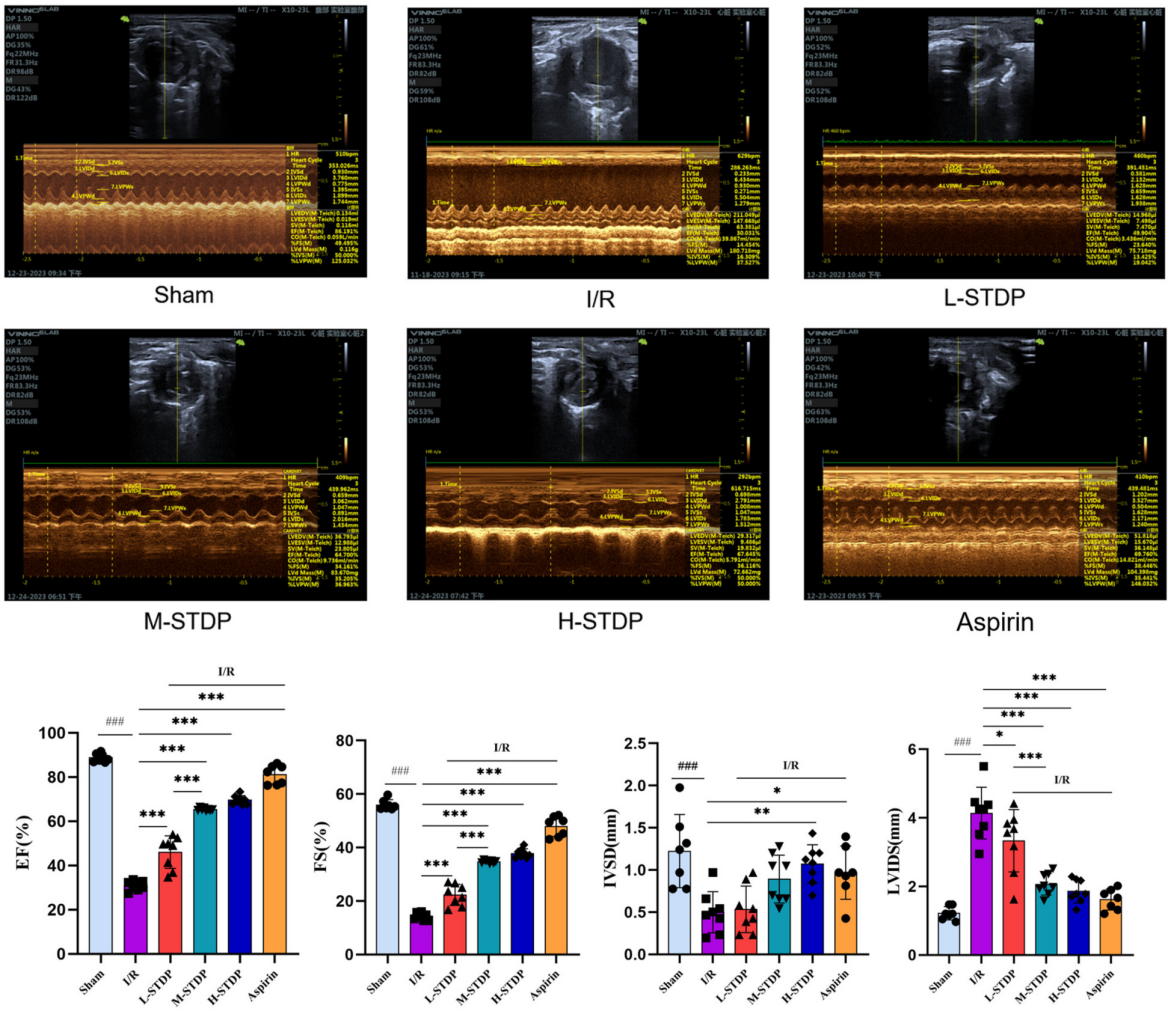
Comparison of M-mode ultrasound images and EF, FS, IVSD, LVIDS of left ventricle in mice after MIRI. Data are represented as mean ± SD. ^###^*P* < 0.001. *P < 0.05; **P < 0.01; ***P < 0.001.

Echocardiographic assessment of mice indicated significant changes between the sham, model, and therapy groups. In the sham group, the left ventricular motion curves showed significant movement amplitudes in both the front and posterior walls, indicating symmetry. In contrast, the model group's coefficients showed a significant increase of the ventricular chamber and thinning of the ventricular anterior wall. The left ventricular motion curves revealed a considerable reduction in anterior wall movement amplitudes. When compared to the model group, mice in the positive control and traditional Chinese medicine groups had thicker ventricular anterior walls and enlarged ventricular cavities. Compared to the Sham group, mice in the ischemia/reperfusion (I/R) group showed significant decreases in ejection fraction (EF), fractional shortening (FS), and interventricular septum diameter in diastole (IVSD) (*P* < 0.01), as well as a marked increase in left ventricular internal dimension in systole (LVIDS) (*P* < 0.01). These data point to the successful creation of the myocardial ischemia-reperfusion damage model, with a significant loss in heart function following surgery. Compared to the I/R group, mice in the low-dose (L-STDP), medium-dose (M-STDP), and high-dose (H-STDP) Shexiang Tongxin Dropping Pills groups, as well as the Aspirin group, showed significant increases in EF, FS, and IVSD (*P* < 0.01), whereas LVIDS decreased significantly (*P* < 0.01). Importantly, STDP-mediated cardioprotection demonstrated a dose-dependent response, with higher doses yielding progressively greater improvements in functional parameters (*P* < 0.001 for L-STDP vs. M-STDP). These findings suggest that the medication can significantly enhance cardiac function after myocardial reperfusion injury (Figure 1).

### 3.2 Myocardial tissue TTC staining and myocardial infarction ratio in mice

**Figure 2:**
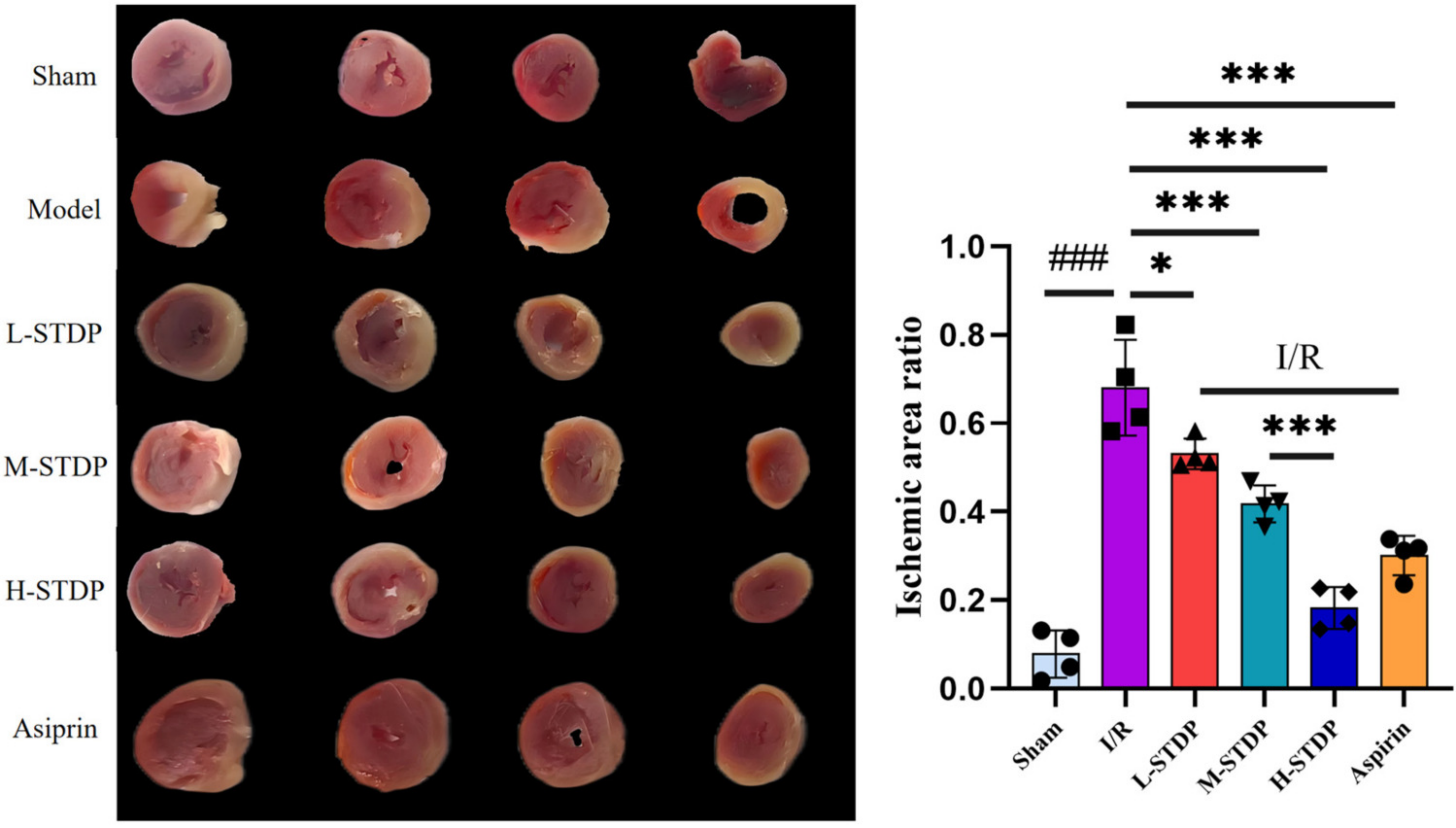
Comparison of TTC staining images and statistical maps of mice myocardial tissue after MIRI. Data are represented as mean ± SD. ^###^*P* < 0.001. *P < 0.05; **P < 0.01; ***P < 0.001.

The results of TTC staining revealed that the sham group showed almost no myocardial infarction, while the model group exhibited significantly increased myocardial infarction compared to the sham group (*P* < 0.05). In contrast to the model group, all STDP-treated groups and the aspirin group demonstrated a reduction in the myocardial infarction ratio (*P* < 0.05). The effect of STDP administration was dose-dependent, with the high-dose group proving more effective than the medium-dose group (*P* < 0.001 for M-STDP vs. H-STDP). These findings suggest that the drug can significantly improve the ischemic infarct area of myocardial tissue after myocardial reperfusion injury (Figure 2).

### 3.3 Effects on myocardial pathological morphology

**Figure 3:**
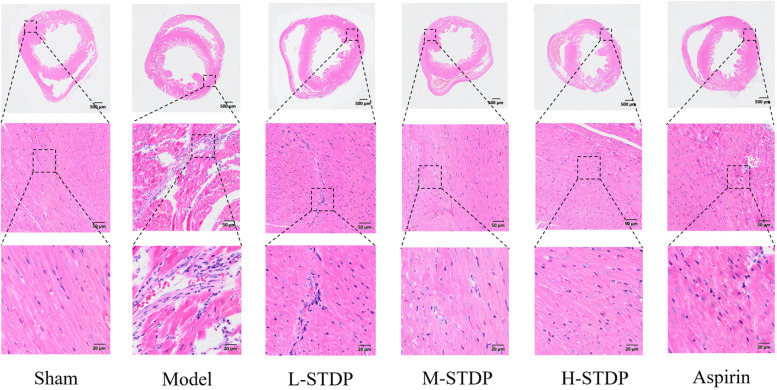
Effect of Shexiang Tongxin Dripping Pills on myocardial pathological changes in MIRI mice.

In the model group, there was obvious interstitial edema in the heart, which was accompanied by severe necrosis of cardiomyocytes, loss of nuclei, red staining of the cytoplasm, and substantial neutrophil infiltration around the dead cells. Myofilament disruptions and myofiber degradation were also observed. In comparison to the model group, the positive drug group had much less interstitial edema in the myocardium, with more ordered arrangement of myofibrils and localized moderate vascular dilatation and congestion. In the medium and high-dose groups of Shexiang Tongxin Dropping Pills (STDP), there was also considerable reduction of cardiac interstitial edema, with cleanly ordered myofibrils and infrequent infiltration of inflammatory cells and minor foci of necrosis. In the low-dose STDP group, minor interstitial edema in the heart was detected, with spatially ordered arrangement of myofibrils and tiny patches of cardiomyocyte necrosis (Figure 3).

### 3.4 Impact of Shexiang Tongxin Dropping Pills on ROS levels in myocardial tissue of MIRI mice

**Figure 4:**
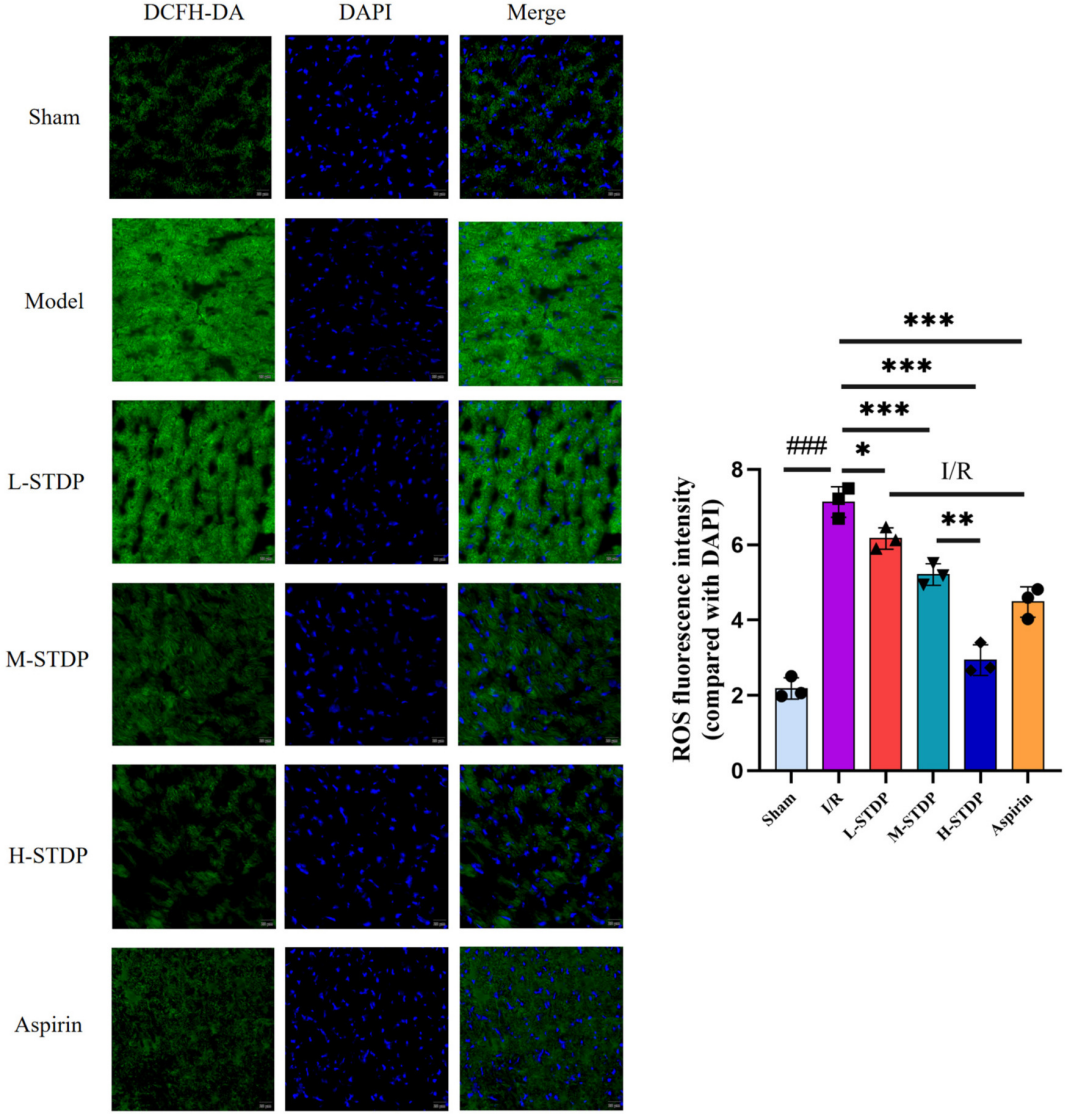
Effect of Shexiang Tongxin Dripping Pills on ROS levels in myocardial tissue of MIRI mice (×200). Data are represented as mean ± SD. ^###^*P* < 0.001. *P < 0.05; **P < 0.01; ***P < 0.001.

Mice in the model group had significantly higher ROS levels in their myocardial tissue compared to the sham group *(P* < 0.01), whereas mice in the L-STDP, M-STDP, H-STDP, and Aspirin groups had significantly lower ROS levels (*P* < 0.05). Furthermore, the improvement in ROS levels in cardiomyocytes exhibited a dose-dependent relationship, becoming more optimal with increasing drug dosage (*P* < 0.01 for M-STDP vs. H-STDP). These results indicate that the medication can significantly ameliorate oxidative stress damage in cardiomyocytes following myocardial reperfusion injury (Figure 4).

### 3.5 Detection of cardiomyocyte apoptosis by TUNEL staining

**Figure 5:**
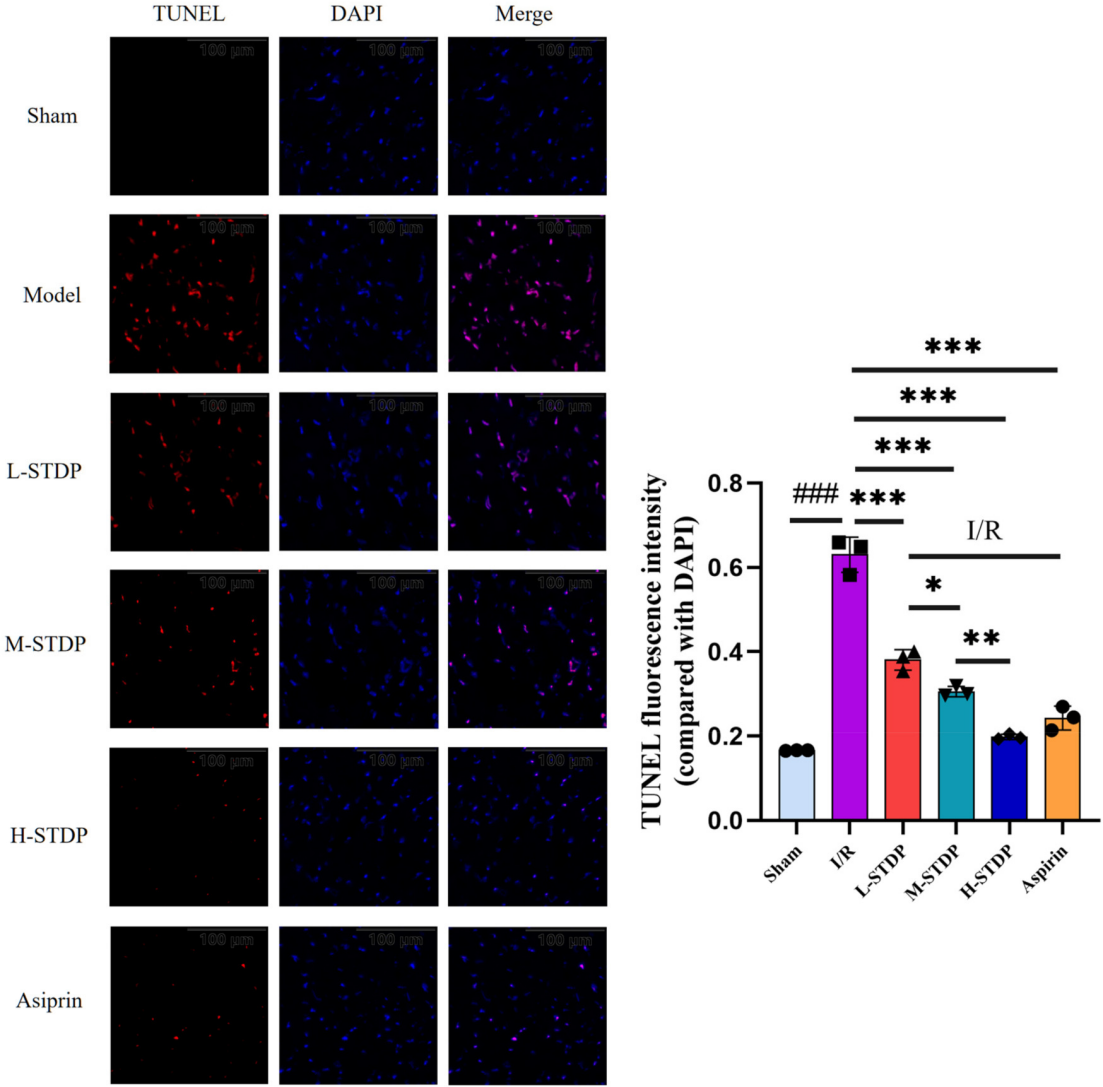
Effect of Shexiang Tongxin Dripping Pills on TUNEL levels in myocardial tissue of MIRI mice (×200). Data are represented as mean ± SD. ^###^*P* < 0.001. *P < 0.05; **P < 0.01; ***P < 0.001.

TUNEL staining demonstrated a marked increase in cardiomyocyte apoptosis rates in the model group compared to the sham group (*P* < 0.001). Pretreatment with STDP and aspirin significantly reduced apoptosis indices in all treatment cohorts relative to the model group (*P* < 0.001). Notably, these therapeutic effects exhibited a dose-dependent pattern, with higher-dose regimens demonstrating superior efficacy over lower-dose interventions(*P* < 0.05 for L-STDP vs. M-STDP, *P* < 0.01 for M-STDP vs. H-STDP). Collectively, these findings indicate that the pharmacological intervention effectively alleviates post-reperfusion cardiomyocyte apoptosis (Figure 5).

### 3.6 Compound-target-disease protein interaction network and pathway enrichment analysis

**Figure 6:**
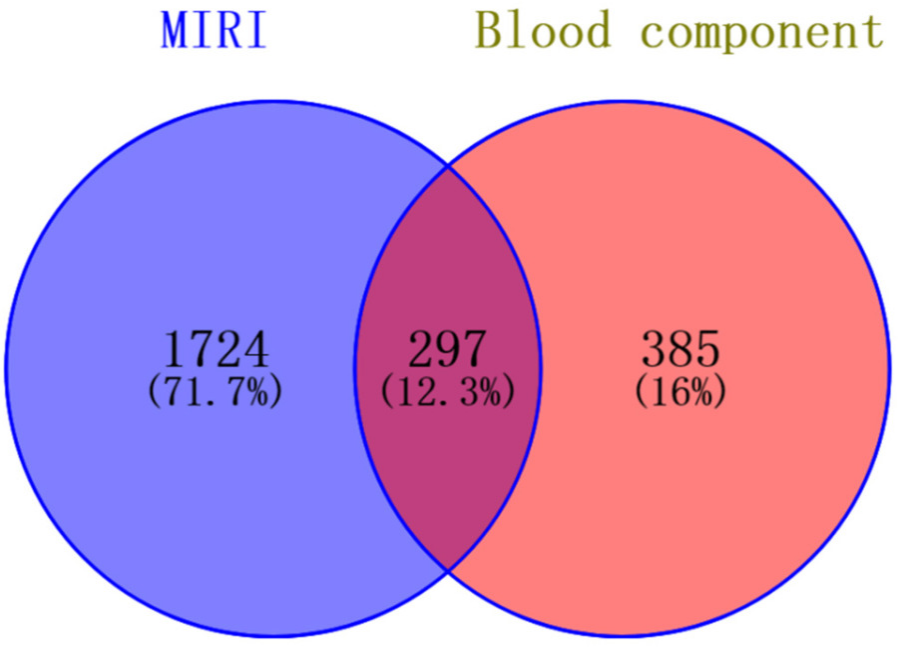
Intersection diagram of blood components and disease targets of Shexiang Tongxin Dripping Pills.

**Figure 7:**
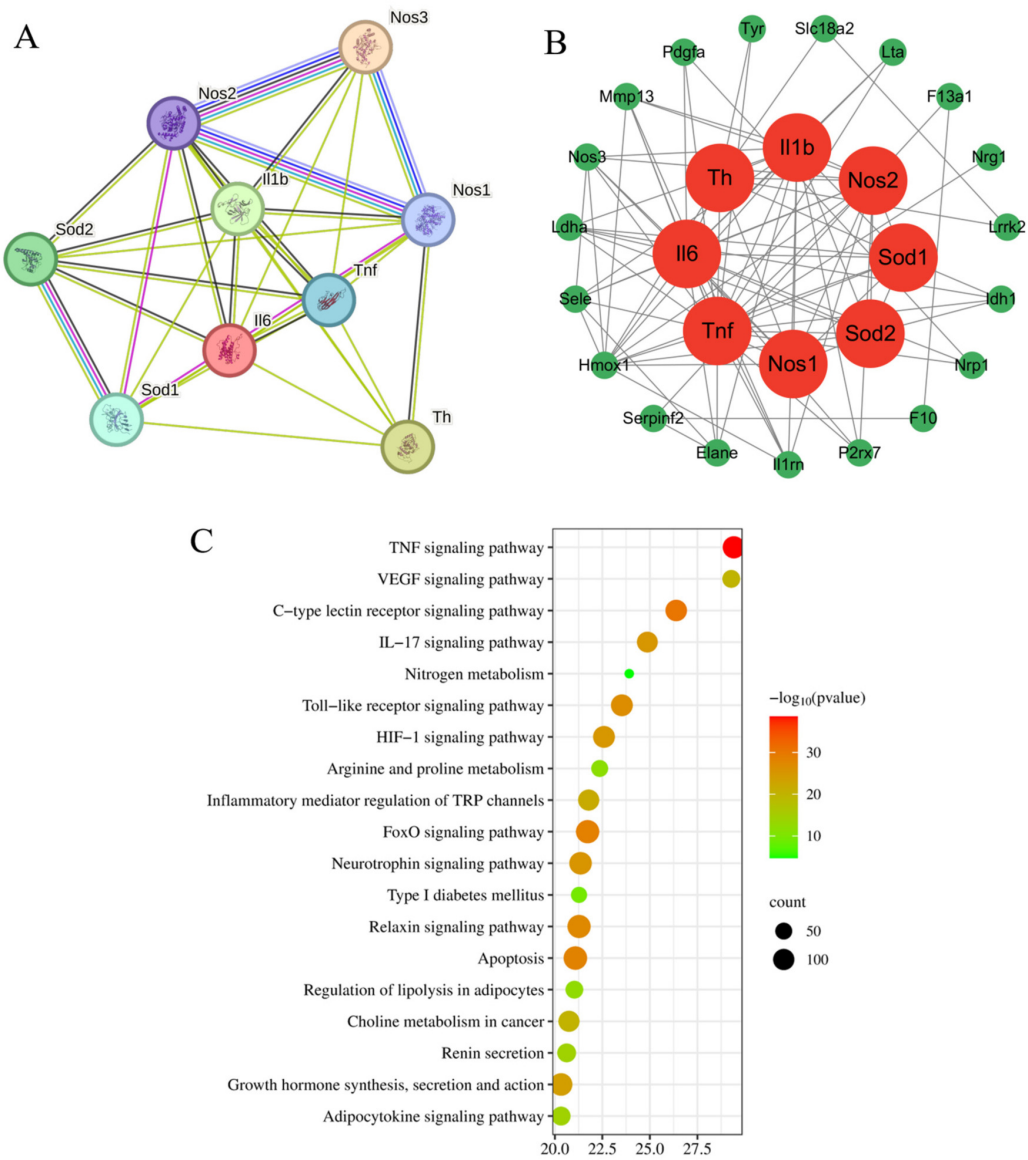
Target protein interaction network diagram and KEGG analysis enrichment pathway diagram. **(A)** Target protein interaction network; **(B)** Core target protein enrichment map; **(C)** KEGG pathway enrichment map.

Drawing upon our team's previous research endeavors, we have successfully identified 30 blood-borne constituents in the Shexiang Tongxin Dropping Pills from the blood samples of normal rats, among which cryptotanshinone is included. The specific information and names of these constituents are detailed in Tables 2, 3. All corresponding targets for these blood-borne components were retrieved by searching the BATMAN-TCM database (49), resulting in 682 unique targets after deduplication. Additionally, 2021 unique targets were obtained by searching the GeneCards database using keywords like “myocardial ischemia-reperfusion,” “ischemia-reperfusion,” “myocardial ischemia-reperfusion injury,” and “ischemia-reperfusion injury,” after deduplication. Using Venny 2.1.0, 297 targets were found to be common between the blood-borne components and disease targets (Figure 6). By building a target protein interaction network and conducting KEGG enrichment analysis (Figure 7), it was discovered that the key targets at the center of this network are SOD, IL-6, IL-1β, and TNF-α. The proposed mechanism of action pathways may involve TNF-related inflammation and oxidative stress pathways.

**Table 2:** The results of UHPLC-Q TOF-MS analysis and identification of the blood-borne components of Shexiang Tongxin Dropping Pills in positive ion mode.

Serial number	Retention time (min)	Precursor Ion	Measured molecular weight m/z	Error (ppm)	Molecular formula	Compound name	MS/MS
1	0.760	[M + H]^+^	126.0218	1.11	C_2_H_7_NO_3_S	Taurine	108.0127, 96.9966, 80.962
2	0.794	[M + H]^+^	184.0971	−1.52	C_9_H_13_NO_3_	L-Adrenaline	166.0868, 151.0692, 107.0492
3	0.956	[M + H]^+^	136.0617	0.53	C_5_H_5_N_5_	Adenine	119.0394, 110.0344, 91.0545, 55.0288
4	1.090	[M + H]^+^	137.0456	1.37	C_5_H_4_N_4_O	Hypoxanthine	119.0350, 94.0397, 82.0394
5	2.711	[M + H]^+^	177.1024	−0.91	C_10_H_12_N_2_O	5-Hydroxytryptamine	160.0759, 132.0804, 115.0538
6	8.341	[M + H]^+^	403.2490	−2.73	C_24_H_34_O_5_	Gamabufotalin	385.2376, 367.2284, 349.2167, 253.1960
7	13.174	[M + H]^+^	417.2276	−1.76	C_24_H_32_O_6_	Arenobufagin	399.2150, 175.0762, 147.1175, 107.0860, 81.0707
8	13.805	[M + H]^+^	417.2281	−2.24	C_24_H_32_O_6_	Desacetylcinobufotalin	399.2160, 175.0759, 147.1170, 107.0854, 81.0705
9	14.903	[M + H]^+^	403.2488	−2.23	C_24_H_34_O_5_	Telocinobufagin	385.2372, 349.2166, 253.1961
10	16.082	[M + H]^+^	500.3039	0.27	C_26_H_45_NO_6_S	Taurochenodeoxycholic acid	482.2924, 464.2827
11	16.473	[M + H]^+^	445.2589	−0.98	C_26_H_36_O_6_	Bufotalin	385.2376, 367.2277, 349.2171
12	17.603	[M + H]^+^	459.2376	0.28	C_26_H_34_O_7_	Cinobufotalin	417.2265, 363.1957, 345.1844, 201.1635
13	17.711	[M + H]^+^	385.2386	1.39	C_24_H_32_O_4_	Resibufogenin	385.2368, 367.2263, 349.2162
14	18.134	[M + H]^+^	387.2538	−2.10	C_24_H_34_O_4_	Bufalin	351.2325, 305.2261, 255.2119, 215.1805
15	18.809	[M + H]^+^	443.2437	−2.00	C_26_H_34_O_6_	Cinobufagin	401.2323, 365.2116, 347.2010, 215.1800
16	19.431	[M + H]^+^	399.2174	−2.00	C_24_H_30_O_5_	Bufogenin	381.2069, 363.1941, 353.2051, 257.1906
17	22.138	[M + H]^+^	277.0870	−3.90	C_18_H_12_O_3_	Tanshinone I	249.0927, 231.0814, 193.1022, 178.0786
18	22.849	[M + H]^+^	297.1501	−4.98	C_19_H_20_O_3_	Cryptotanshinone	282.1238, 279.1390, 251.1447
19	23.905	[M + H]^+^	295.1342	−4.50	C_19_H_18_O_3_	Tanshinone IIA	277.1240, 266.0941, 249.1286, 235.0757

**Table 3:** The results of UHPLC-Q TOF-MS analysis and identification of the blood-borne components of Shexiang Tongxin Dropping Pills in negative ion mode.

Serial number	Retention time (min)	Precursor ion	Measured molecular weight m/z	Error (ppm)	Molecular formula	Compound name	MS/MS
20	2.377	[M-H]^−^	197.0452	1.76	C_9_H_10_O_5_	Danshensu	179.0374, 135.0473, 123.0466, 72.9945
21	3.411	[M-H]^−^	137.0245	−0.60	C_7_H_6_O_3_	Protocatechuic aldehyde	119.0148, 108.0228, 92.0279, 81.0360, 65.0050, 53.0404
22	8.709	[M + COOH]^−^	845.4879	2.97	C_42_H_72_O_14_	Ginsenoside Rg_1_	799.4838, 637.4322, 475.3789, 161.0478, 89.0252
23	9.325	[M + COOH]^−^	991.5480	0.32	C_48_H_82_O_18_	Ginsenoside Re	945.5444, 783.4884, 637.4324, 475.3797
24	10.652	[M-H]^−^	717.1474	−1.80	C_36_H_30_O_16_	Salvianolic acid B	519.0947, 321.0419, 295.0626
25	14.714	[M-H]^−^	498.2903	−1.64	C_26_H_45_NO_6_S	Tauroursodeoxycholic acid	432.3111, 372.2901, 124.0087
26	15.218	[M + COOH]^−^	1,153.6013	−0.14	C_54_H_92_O_23_	Ginsenoside Rb_1_	1,107.5942, 945.5392, 783.4862, 579.4510
27	17.021	[M-H]^−^	1,077.5848	1.39	C_53_H_90_O_22_	Ginsenoside Rb_2_	945.5439, 915.5296, 783.4877, 621.4361
28	17.389	[M + COOH]^−^	991.5478	0.52	C_48_H_82_O_18_	Ginsenoside Rd	945.5440, 783.4897, 621.4367,459.3831
29	17.891	[M + COOH]^−^	683.4384	−1.19	C_36_H_62_O_9_	Ginsenoside Rh_1_	637.4326, 475.3800, 179.0579, 161.0477
30	20.110	[M + COOH]^−^	829.4941	1.68	C_42_H_72_O_13_	Ginsenoside Rg_3_	783.4911, 621.4371, 459.3846, 161.0477, 101.0255

### 3.7 Effects of Shexiang Tongxin Dropping Pills on mRNA expression of Key targets in inflammatory pathways in the heart tissue of MIRI mice

**Figure 8:**
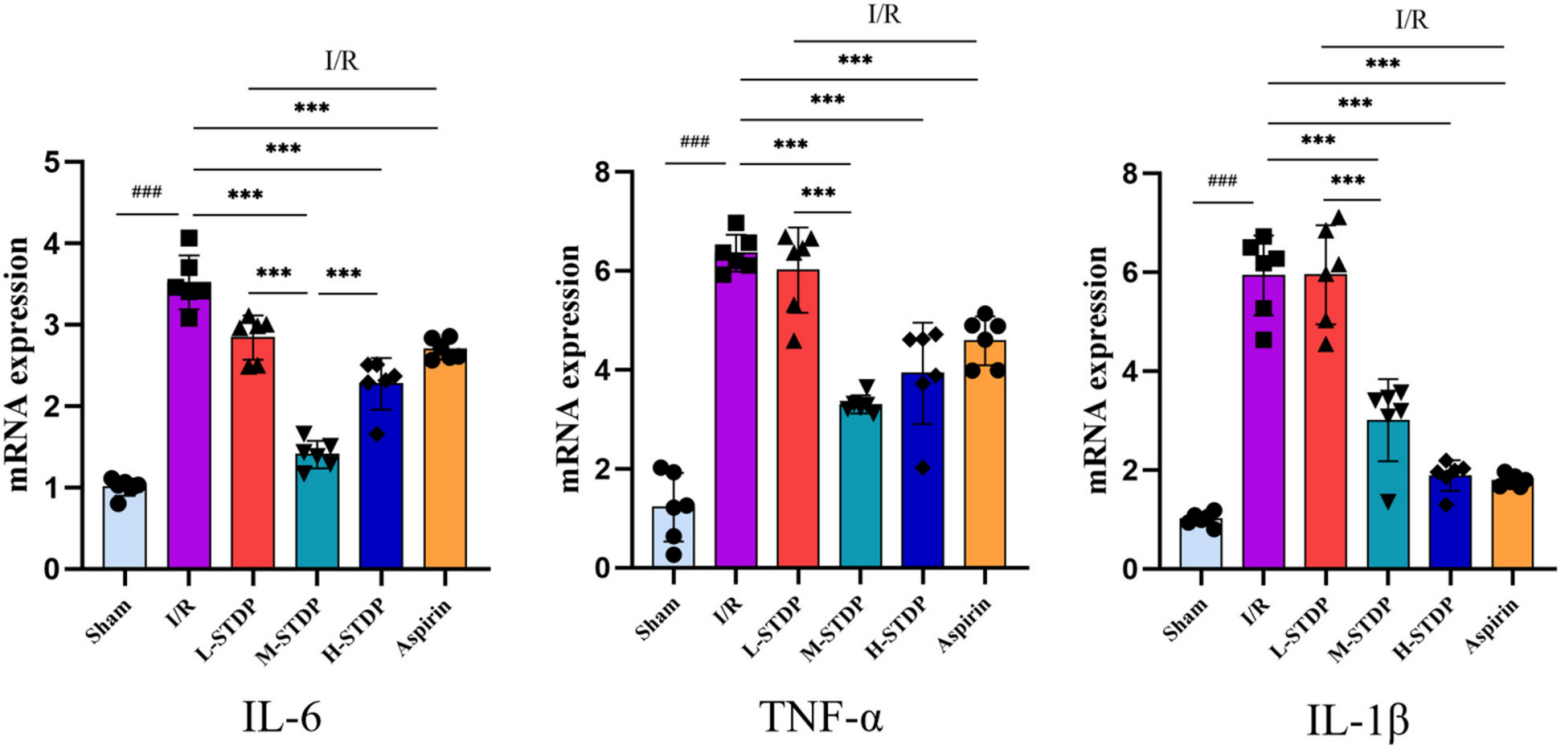
Effects of Shexiang Tongxin Dripping Pills on the expression of IL-6, TNF-α and IL-1β mRNA in the heart of MIRI mice. Data are represented as mean ± SD. ^###^*P* < 0.001. *P < 0.05; **P < 0.01; ***P < 0.001.

The model group had considerably higher mRNA expression levels of IL-6, TNF-α, and IL-1β in cardiac tissue than the control group, according to qRT-PCR data, with statistically significant differences (*P* < 0.05). The Aspirin group, high-dose Shexiang Tongxin Dropping Pills group, and medium-dose Shexiang Tongxin Dropping Pills group all displayed statistically significant (*P* < 0.05) reductions in mRNA expression levels of IL-6, TNF-α, and IL-1β in heart tissue when compared to the model group. Moreover, the improvement in the inflammatory status of the myocardial tissue showed a dose-dependent effect, becoming more pronounced with increasing dosage (*P* < 0.001 for L-STDP vs. M-STDP). These results suggest that Shexiang Tongxin Dropping Pills can reduce inflammatory reactions in MIRI mice's heart tissue (Figure 8).

### 3.8 Effects of Shexiang Tongxin Dropping Pills on serum oxidative stress and inflammatory-related protein expression in mice

**Figure 9:**
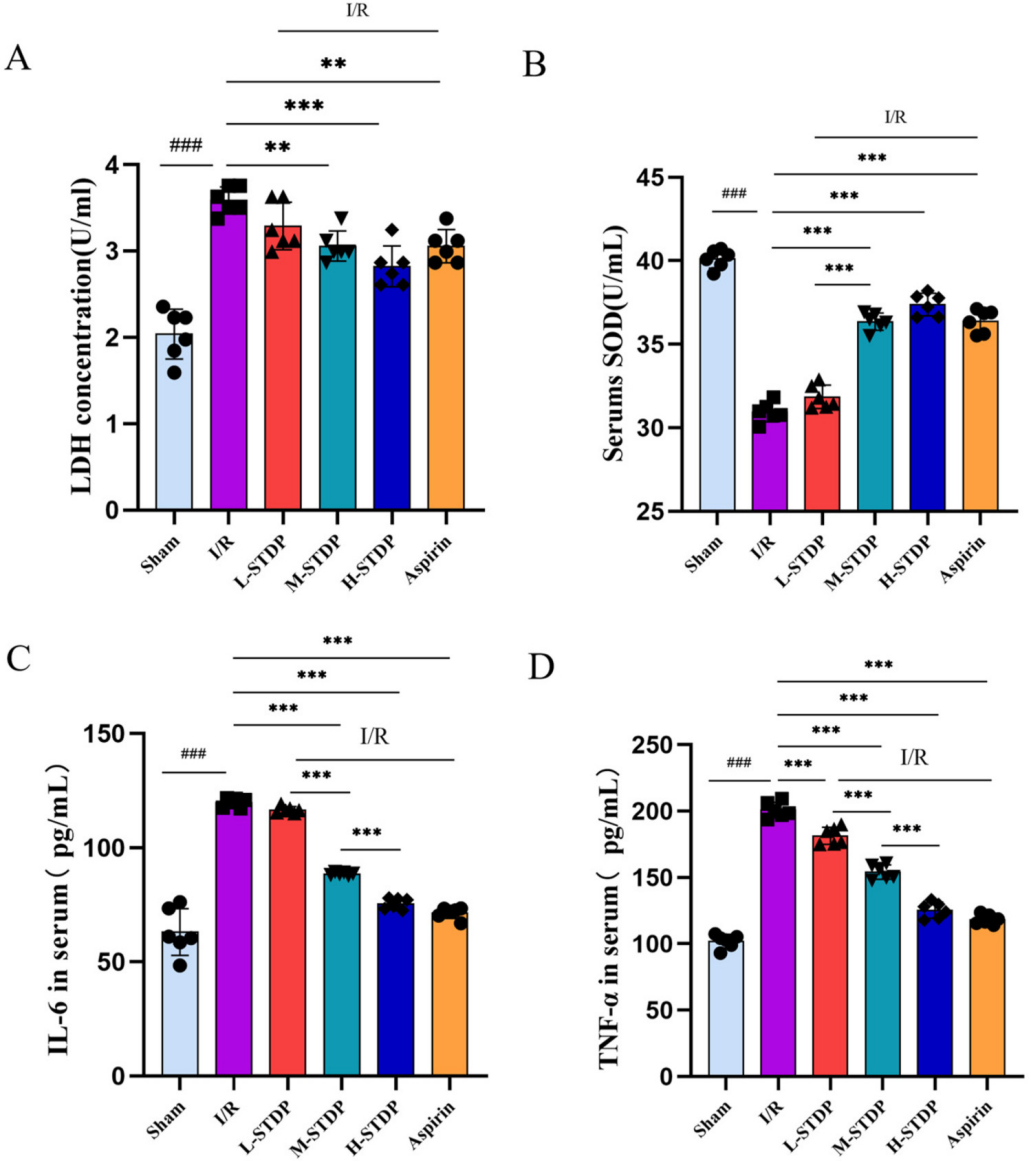
Effects of Shexiang Tongxin Dripping Pills on the expression of SOD, LDH, IL-6 and TNF-α in serum of MIRI mice. **(A)** LDH content in serum of mice in each group; **(B)** SOD content in serum of mice in each group; **(C)** IL-6 content in serum of mice in each group; **(D)** TNF-α content in serum of mice in each group. Data are represented as mean ± SD. ^###^*P* < 0.001. *P < 0.05; **P < 0.01; ***P < 0.001.

Compared with the sham group, mice in the model group exhibited significantly elevated levels of LDH (*P* < 0.01) and significantly decreased levels of SOD (*P* < 0.01) in their serum, which are key targets in the oxidative stress pathway. In contrast, when compared to the model group, mice in the Shexiang Tongxin Dropping Pills group showed marked decreases in LDH levels (*P* < 0.01) and significant increases in SOD levels (*P* < 0.01) in their serum. Additionally, compared with the sham group, mice in the model group had significantly elevated levels of TNF-α and IL-6 (*P* < 0.01), which are key targets in the inflammatory pathway. However, when compared to the model group, mice in the Shexiang Tongxin Dropping Pills group exhibited significant decreases in TNF-α and IL-6 levels (*P* < 0.01) in their serum. Notably, STDP exhibited marked dose-dependent cardioprotection, demonstrating progressive suppression of oxidative stress and inflammatory mediators with successive dose escalation. Compared to low-dose treatment (L-STDP), medium-dose administration (M-STDP) significantly attenuated myocardial SOD levels (*P* < 0.001), while high-dose intervention (H-STDP) induced stepwise reductions in both TNF-α and IL-6 concentrations compared to lower dosage groups (*P* < 0.001 for all comparisons). This graded pharmacological response pattern mechanistically establishes that STDP confers protection against myocardial ischemia-reperfusion injury through coordinated attenuation of oxidative damage and stepwise inhibition of inflammatory cascades, directly correlating with therapeutic concentration (Figure 9).

### 3.9 Impact of Shexiang Tongxin Dropping Pills on the expression of inflammation-related proteins in mouse myocardial tissue

**Figure 10:**
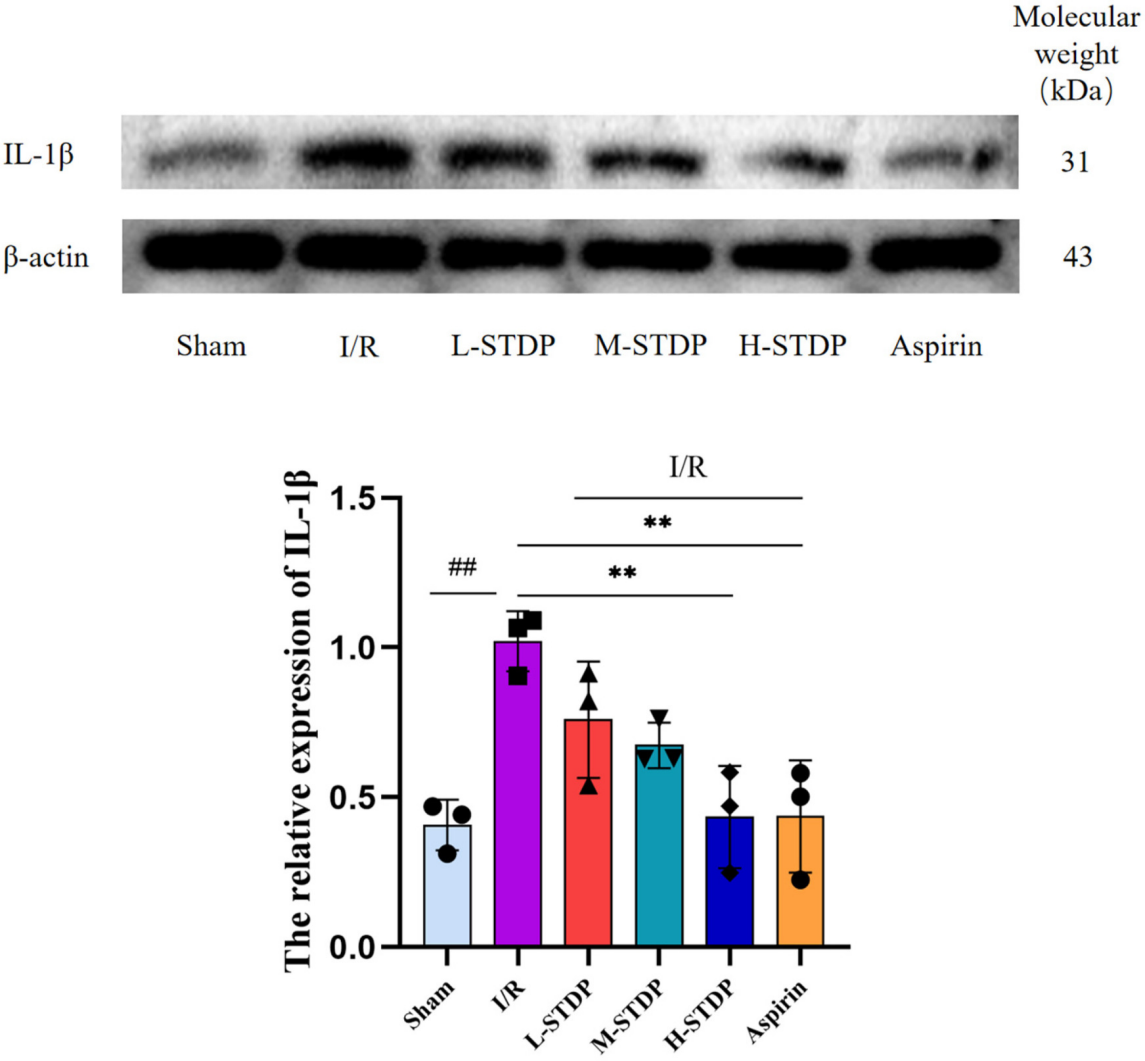
Effect of ShexiangTongxin Dripping Pills on the expression level of IL-1β protein in myocardial tissue of MIRI mice.Data are represented as mean ± SD. ^##^*P* < 0.01. *P < 0.05; **P < 0.01; ***P < 0.001.

When compared to the sham group, the model group exhibited a notable increase in IL-1β protein expression in myocardial tissue. Conversely, when compared to the model group, both the H-STDP and Aspirin groups showed a significant decrease in IL-1β protein expression in myocardial tissue (*P* < 0.05). These findings suggest that Shexiang Tongxin Dropping Pills inhibit the further exacerbation of inflammatory responses (Figure 10).

## 4 Discussion

Reperfusion is an essential process in the treatment of myocardial ischemia, aimed at improving myocardial blood supply. However, it is accompanied by a series of complex pathophysiological responses, including oxidative stress, inflammatory responses, calcium overload, mitochondrial dysfunction, and ferroptosis. Among these, oxidative stress is a key driver of reperfusion injury (50). Currently, traditional Chinese medicine (TCM) clinically employs organic compounds such as flavonoids and natural compounds from Chinese herbs to prevent and treat myocardial ischemia-reperfusion injury (MIRI) by inhibiting oxidative stress, inflammatory responses, and apoptosis. However, the underlying mechanisms remain incompletely understood (51, 52). In Western medicine, propofol, sevoflurane, etomidate, statins, and other drugs are used to alleviate MIRI by regulating pathways such as MAPK/NF-*κ*B to suppress inflammatory cytokines and oxidative stress. Yet, these treatments have drawbacks such as poor safety, strong side effects, and uninvestigated mechanistic targets (53).

Previous research by our research group found that Shexiang Tongxin Dropping Pills (STDP) promote macrophage polarization and angiogenesis through the PI3 K/Akt/mTORC1 pathway, thereby alleviating coronary microvascular dysfunction following myocardial ischemia-reperfusion (29). Therefore, this study focuses on STDP. The major blood components of STDP include 31 compounds such as hypoxanthine, bufalin, tanshinone I, protocatechuic aldehyde, ginsenoside Rg1, and salvianolic acid B. Using network pharmacological methods, this study screened potential targets for these 31 compounds and identified 297 common targets through intersection with targets related to myocardial ischemia-reperfusion injury. Protein-protein interaction (PPI) analysis of these common targets was conducted using the STRING database to construct a PPI network. Based on network topology parameters, eight core targets were selected. Among them, TNF and IL can inhibit the release of inflammatory cytokines TNF-α and IL-1β, and reduce myocardial cell apoptosis and other damage through SOD-mediated inhibition of oxidative stress, thereby improving myocardial ischemia-reperfusion injury (54). By constructing and analyzing the “STDP blood components–targets–pathways” network, the top five key core targets identified were SOD, TNF, IL-1β, IL-6, and NOS. The top five Kyoto Encyclopedia of Genes and Genomes (KEGG) pathways included the TNF signaling pathway, VEGF signaling pathway, C-type lectin receptor signaling pathway, IL-17 signaling pathway, and nitrogen metabolism pathway. These targets and pathways are associated with multiple physiological processes such as inflammation and oxidative stress, demonstrating high connectivity centrality in network topology parameter analysis. This suggests that the potential mechanism of STDP in protecting the myocardium from reperfusion injury is highly correlated with these targets and pathways.

Declines in myocardial diastolic and systolic function directly impact cardiac pumping and hemodynamic functions. Left ventricular ejection fraction (LVEF) and left ventricular fractional shortening (LVFS) are the most commonly used clinical indicators for monitoring cardiac function, accurately reflecting changes in cardiac function and hemodynamics. Hematoxylin and eosin (HE) staining allows observation of myocardial fibers, myocytes, and inflammatory cells (55). A foundational study identified elevated oxidative stress during blood reperfusion as the cardinal pathological alteration underlying Myocardial Ischemia-Reperfusion Injury (MIRI). During MIRI reperfusion, cardiovascular endothelial cells experience impaired mitochondrial function, enhanced catecholamine auto-oxidation, and a dramatic surge in neutrophil respiratory activity, all contributing to reactive oxygen species (ROS) generation. Concurrently, diminished activity and expression of antioxidant enzyme transporters compromise myocardial defense mechanisms. The resultant ROS accumulation disrupts intracellular redox homeostasis, precipitating structural damage to cardiomyocytes, metabolic dysfunction, and apoptosis. Emerging evidence underscores that mitochondrial ROS overproduction, energy metabolic imbalance, and cellular infrastructure destabilization serve as critical determinants of aberrant cardiomyocyte apoptosis in MIRI (56–59). LDH levels reflect the degree of myocardial cell damage, with higher levels indicating increased intracellular generation of oxygen free radicals and their damage to cells (60). Based on our research group's previously established method for preparing mouse models of myocardial ischemia-reperfusion injury (38), this study constructed a MIRI mouse model by ligating the left anterior descending coronary artery (LAD). The findings revealed a dose-dependent cardioprotective effect of STDP pretreatment in a mouse model of myocardial ischemia-reperfusion injury (MIRI). Specifically, high-dose STDP selectively enhanced the recovery of cardiac function, alleviated oxidative stress, and suppressed the expression of inflammatory cytokines. In the MIRI model, the successful induction of ischemic injury was confirmed by significant reductions in left ventricular ejection fraction (LVEF) and left ventricular fractional shortening (LVFS), as well as histopathological features such as myocardial edema, fiber disarray, inflammatory infiltration, elevated levels of reactive oxygen species (ROS), and increased rates of cardiomyocyte apoptosis. STDP pretreatment dose-dependently ameliorated these perturbations: high-dose STDP notably restored LVEF and LVFS, decreased myocardial ROS accumulation, reduced the rate of cardiomyocyte apoptosis, and enhanced superoxide dismutase (SOD) activity, which were associated with preserved myocardial ultrastructure and improved contractile-relaxation performance. At the molecular level, higher-dose STDP exhibited superior efficacy in downregulating mRNA expression of pro-inflammatory cytokines (TNF-α, IL-6, and IL-1β) and suppressing IL-6 and TNF-α protein levels, suggesting its capacity to disrupt transcriptional and translational inflammatory cascades. However, while STDP reduced serum lactate dehydrogenase (LDH) and myocardial IL-1β protein expression, these effects were not dose-dependent, implying distinct regulatory mechanisms. The anti-inflammatory properties of STDP likely involved inhibition of NF-*κ*B signaling, thereby attenuating TNF-α and IL-6 synthesis (61), whereas its antioxidant effects were mediated through SOD upregulation, potentially via activation of the Nrf2/ARE pathway (62). The lack of dose dependency in IL-1β protein modulation—despite reduced mRNA levels—may reflect post-transcriptional regulation, such as inflammasome-dependent IL-1β maturation (e.g., NLRP3 activation) (63), which remained unaltered by STDP dosing. Similarly, the dose-independent reduction in LDH, a marker of membrane integrity, suggested that STDP stabilized cardiomyocyte membranes through mechanisms independent of oxidative-inflammatory crosstalk, possibly involving direct membrane repair or calcium homeostasis (64). The superior efficacy of STDP in restoring redox balance (via ROS reduction and SOD restoration) and suppressing TNF-α/IL-6 expression highlighted the therapeutic potential of dose optimization. The dose-dependent effects on these parameters suggested that higher STDP concentrations more potently engaged critical signaling nodes, such as Nrf2-driven antioxidant defenses and NF-*κ*B inhibition. Conversely, the absence of dose-responsive effects on IL-1β protein and LDH underscored the multifactorial nature of MIRI pathophysiology, where parallel pathways [e.g., mitochondrial permeability transition (65), inflammasome activation (66)] may dominate certain aspects of injury progression. These findings positioned STDP as a dual-target agent capable of mitigating oxidative and inflammatory injury in MIRI, albeit with mechanistic selectivity. To further enhance the translational relevance of these findings, future studies evaluating STDP's pharmacokinetic profile and long-term safety would provide critical insights into its therapeutic window and clinical applicability. Additionally, expanding mechanistic investigations to explore STDP's interactions with other regulators—such as MAPK or autophagy-related signaling pathways—would help refine our understanding of its broader cardioprotective network and potential synergies with existing therapies. Employing multi-omic approaches in subsequent work would further delineate STDP's global targets and clarify its role in modulating cross-pathway dynamics, particularly in combination with ischemic preconditioning or adjunctive reperfusion strategies. In conclusion, our work established that STDP pretreatment alleviated MIRI through dose-dependent modulation of oxidative and inflammatory pathways, with higher doses offering enhanced cardioprotection. By delineating the scope and limits of its efficacy, our findings provided a mechanistic foundation for optimizing STDP-based therapies in ischemic heart disease, while emphasizing the need for integrative approaches to address the complexity of MIRI pathophysiology.

## 5 Conclusion

In summary, Shexiang Tongxin Dropping Pills demonstrate efficacy in ameliorating oxidative stress damage induced by myocardial ischemia-reperfusion in mice, enhancing antioxidant capacity, mitigating myocardial tissue damage, improving cardiac function, and protecting myocardial tissue. The mechanism of action may be attributed to the anti-inflammatory and anti-oxidative stress properties of individual herbs within its compound formulation, such as Salvia miltiorrhiza. Moving forward, our research team aims to further explore the correlation between the spatial distribution of components and their mechanisms of action, delving deeply into the specific processes of functional components and pathway regulation. The ultimate objective is to systematically unveil the precise regulatory mechanisms of the efficacious components.

## Data Availability

The datasets presented in this study can be found in online repositories. The names of the repository/repositories and accession number(s) can be found in the article/Supplementary Material.
