# Supplementary material for: Shexiang Tongxin Dripping Pills regulates SOD/TNF-α/IL-6 pathway to inhibit inflammation and oxidative stress to improve myocardial ischemia-reperfusion injury in mice
Source: Front Cardiovasc Med. 2025 Jun 12;12:1571925. doi: 10.3389/fcvm.2025.1571925 (PMC12198137; doi:10.3389/fcvm.2025.1571925)
Supplement: Supplementary file 1 [file Image1.pdf]

Supplementary Material

1.1 Supplementary Figures

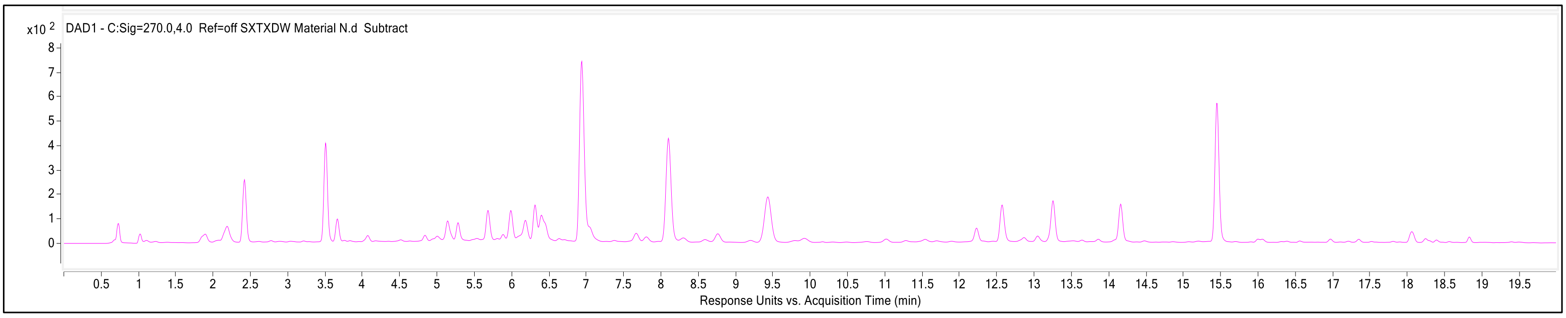

Supplementary Figure 1. Chromatogram of Shexiang Tongxin Dripping Pills at 270 nm.
